# Nursing in Ghana: A Search for Florence Nightingale in an African City

**DOI:** 10.1155/2016/9754845

**Published:** 2016-03-24

**Authors:** Samuel Adu-Gyamfi, Edward Brenya

**Affiliations:** Department of History and Political Studies, Kwame Nkrumah University of Science and Technology (KNUST), Kumasi, Ghana

## Abstract

Nursing in Ghana is a crucial subject that permeates almost every issue in the society especially the field of hospital care. To a large extent, the frontiers of nursing have expanded since the time of Florence Nightingale. Globally some studies have been done to study nursing icons like her. The values in nursing practice however continue to preoccupy our minds. The need to accentuate the gains made by historical figures in nursing in present times as well as the nature of interactions between practitioners and patients continues to be of paramount concern to many across the globe and Ghana in particular. This study does an analysis of existing literature on Florence Nightingale and the nature of nursing in Ghana from the colonial times. Additionally, it analyzes responses concerning the activities of nurses and their interactions with patients in Kumasi. The varied information has been thematically pieced together to make inferences that are of great interest to nursing practitioners, policy makers, administrators, and educators among others. The findings to the study suggest among other things that the challenges faced by the nursing institution in modern times are similar to those of the earlier period. The study calls for the emulation of the positive ideas of Florence Nightingale to promote the interest of patients, a core objective championed by a revered nurse.

## 1. Introduction and Preconditions for Take-Off

According to Ewusi, the development of western medical system after it had been introduced into the Gold Coast from 1868 was not left to only expatriate medical officers. Referring to Kissieh, our attention is drawn to the fact that, by 1878, the missions and the colonial medical officers had enlisted the help of mail orderlies to bathe and feed the sick, to dress wounds, and to administer drugs to the local population under their medical supervision [1]. The study, however, indicates that nursing in Ghana was firstly a male occupation. However, in 1899, when the first nursing sister arrived in the Gold Coast (Ghana), few ladies began to trickle into the profession of nursing in Ghana [2].

Significantly, the number of nurses and midwives increased side by side with the increase of the medical officers especially from 1952 to 1988. For example, within the period, the population of nurses and midwives increased from 1352 in 1952 to 21,300 in 1988 [3]. The question of equitable distribution of healthcare practitioners and nurses in particular has been looked at by several authors but most importantly the question of effectiveness and efficiency of nursing professionals in Ghana has had little or no attention by social historians of medicine in Ghana. Most importantly, a comparative analysis of the performance and attitudes of nurses in Ghana with the persona, charisma, and achievements of the most celebrated nurse, Florence Nightingale, has not been fully studied.

Of seminal interest is Akiwumi's Higher Education for nurses in which attention is paid to the traditional role of the nurse and the education which prepared her for that role in the 1970s [2].

Akiwumi argued that few of the women had formal education and those who could read and write were given simple lessons in human anatomy and physiology, surgical and medical nursing, and first aid. They were awarded the Director of Medical Service certificate on successful completion of this training and appointed as second-division nurses in the junior civil service. In the field, these nurses were strictly under medical supervision [2]. According to Akiwumi in 1928 midwifery training started in the maternity hospital, then newly built in Korle-Bu. Girls with better education were recruited into this very readily because the nature of the job was more in line with the traditional role women were expected to play in the society [2]. Essentially, the nursing profession or midwifery care was seen by the Europeans as that which could be easily performed by women. This is indicative of the fact that women are generally perceived to be empathetic and would easily take care of people, an ideal this study perceives that falls within Nightingale's philosophy of nursing.

The Nightingale experiment started over a century ago. Before then, nurses received no formal education but rather went through an apprenticeship system, learning about the job and the techniques from older members of the profession. However, upon the establishment of the Nightingale Fund due to the recognition of her impeccable service during the Crimean War, a school was established to formerly train nurses. The school was to train nurses, for a period of one year; nurses usually came into contact with some level of theoretical understanding on diseases. Again, practical training was supervised by ward sisters and doctors [2]. This training was silent on training of nurses on human behaviour, psychology, anthropology and culture, and, to a large extent, sociology of medicine, to enable the trainees to imbibe certain things that will enable them to treat their patients with some greater level of understanding and readings into human behaviour and care. Notwithstanding, it was expected that the ideals of Florence Nightingale (1820–1910) would resonate and again, with further effective supervision especially in these countries in Europe, nurses were not left to pursue their personal predilections per se at the expense of the patient and the rules that govern a particular medical institution or setup. Significantly, the nursing practice emphasized tasks and procedures and these were carried out in a routine manner with not much consideration for the patient, the object of the procedure [2].

According to Akiwumi, before the introduction of rigorous training for nurses, the nurse was always at the behest of the doctor. “The doctor rewarded the nurse who obeyed his instructions and punished the one who did not with humiliation and even the threat of dismissal. He considered a good nurse as the one who could perform the doctors' tasks best” [2]. Akiwumi further argues that under such conditions it was difficult for the nurse to assume responsibility and take initiative or make decisions under emergency situations. The fact was that she was never sure what the boss would say [2].

Significantly, in the twentieth century, social changes increased the complexity of nursing responsibilities and demanded the types of services requiring intellectual competence and a wide range of knowledge [2]. The advancement of knowledge of patients who come to seek medical care and also patients who come to clinics or health facilities with problems which require specialist doctors also required that there were trained specialist nurses to support the work of the physicians. It was required that there was some knowledge in social and behavioural sciences. Long contact hours of nurses with patients, according to Akiwumi, should afford nurses an opportunity to know more about the behaviour of human beings in general than doctors. The nurse should therefore be equipped to direct her actions and verbal expressions on the basis of a sound understanding of human behaviour [2]. The expectation was that this training among others would enhance the performance of the nurse in the general delivery of care to the patients.

It is of great importance to stress that if the nurse has little or no understanding or decides not to apply this knowledge to better the health conditions of patients, she/he may contribute to illness or serve as a disincentive to the other professional medical officers and doctors in particular. Akiwumi further posits that there are different ways of dealing with intense feelings: one is to yield to them, the second to defend against them, and the third to face them directly, work through them, and reduce their power [2]. In all these instances, the nurse is required to manage or live above her personal emotions to be careful not to be tender towards one patient and repulsive towards the other and to some extent their family.

Akiwumi's study proposed six key areas or roles the nurse was supposed to function within at that period. They included the nurse as a technician, the nurse as a provider of care, and the nurse as a team leader. The others include the nurse as a collaborator, the nurse as educator, and the nurse as administrator [2]. Of seminal value to this paper is the nurse as a provider of care: tender loving care. This is a role which was uniquely considered as nursing since the days of Florence Nightingale, the provision of which gives the greatest satisfaction to the nurse and the absence of which causes patients and their families' great distress. According to Akiwumi,

*“the nurse through a greater understanding of man and his behaviour both in health and in illness, should be able to develop sensitivity and understanding for patients; recognize manifestations of anxiety, conflict, fears, and frustrations which underlie a patient's behaviour during illness; establish a nurse-patient relationship which will demonstrate sympathetic interest and concern's for the patient's problems, imagined or real” [2].*

It is pertinent, however, to stress on the life of Florence Nightingale because her ideals permeate the nursing profession even in the twentieth century. Since the twentieth century, the expectation of patients and the larger segment of society is that nurses will be able to perform their duties beyond the nineteenth and twentieth century's standard set by Florence Nightingale.

In the first instance, our attention is turned to a brief history of nursing and then to the story of Florence Nightingale, which is used as the basis to analyze the roles of nurses in the Kumasi metropolis of Ghana. This study also examines the evolution of nursing education in postindependence context of Ghana, nurses and nursing education in Ghana focusing on how it created collaborative opportunities and challenges to the growth of nursing education in Ghana. It also looks at the twenty-first century nursing practice in Ghana and examines the historiography of nursing practice in Ghana to confirm or denounce whether nurses in Ghana have lived above the status of Nightingale. Methodologically, the paper uses responses from nurses within some anonymous selected clinics/hospitals in the Kumasi metropolis of Ghana and patients as data source. The information gathered was analyzed qualitatively under themes and used to draw conclusion. Interestingly, the findings from the study reflect the general perceptions of nurses concerning their own profession, their relationship with patients, and the perception of patients about these nurses. The views shared by both the nurses and the patients and civil society in general have been compared with some of the ideals and performance of Florence Nightingale as a nurse to ascertain whether they have soared above the status quo or are living beneath it.

## 2. A Brief History of Nursing

The development and evolution of the nursing profession are intricately connected to historical influences throughout the ages, beginning in antiquity [4]. In primitive societies, the decision to be a caregiver was often made for a person long before he/she had the ability to make such a choice and, in many societies, the provision of nursing care was a role that was assigned to female members [4]. The reason was because women nurture their own children and were expected to extend the nurturing skill to people who are sick and injured in their communities. Yet in other societies, care of the sick was a role assigned to medicine men, shamans, and other male tribesmen. Because no formal education in the care of the sick was available, the earliest nurses learned their art through oral traditions passed from generation to generation, from observations of others caring for the sick, and many times, through a process of trial and error [4].

Available evidence indicates that nurses first formed themselves into organized groups during the early Christian era. The nursing ideals of charity, service to others, and self-sacrifice were in harmony with the teachings of the early Christian church [4]. Throughout antiquity, the preferable, and often safest, nursing care was provided in one's own home, where one was cared for by family members, clansmen, or friends [4]. Into this setting entered* Florence Nightingale* (said to be the mother of nursing), the woman who would not only reform nursing as it existed at that time, but also laid the foundation for nursing as a profession [4]. The section that follows explores how Florence Nightingale used advocacy as a tool and to identify the value of her conceptual and practical advocacy strategies for the nursing.

## 3. Life of Florence Nightingale

Florence Nightingale was a woman who because of her religious convictions and profound vision of the potential of nursing altered the status of nursing from that of domestic service to that of a profession in the 19th century [5]. Nightingale used personal motivation to create a permanent professional transformation of nursing. Henry Wadsworth Longfellow in 1857 described Nightingale as “the Lady with the Lamp” in his poem.

The Encyclopedia Britannica records that Florence Nightingale was born on 12 May 1820 in Florence and died on 13 August 1910. She was the second daughter born to William Edwards and Frances Nightingale. She was named after the city of her birth. The Nightingales lived a contented lifestyle. They had two homes one located in Lea Hurst in Derbyshire in Central England and the other at Embley Park in warmer Hampshire located in South England [6].

Florence was a bright child and therefore her father gave meticulous attention to her education guiding her through history, philosophy, and literature. She also excelled in mathematics and languages such as French, German, Italian, Greek, and Latin at a tender age. She liked to read great philosophies and to hold in stern political and social conversation with her father [6]. Florence found a great relief in her religious belief; therefore, at the age of sixteen she received one of several “calls from God.” She saw this meticulous calling as decreasing human suffering. To Florence, nursing seemed as an appropriate course to serve both God and humankind. As a result of this, she helped to care for her sick relatives and tenants on the family estates. Her efforts to seek nurse's training were hindered by her family as an inappropriate activity for a woman of her calibre [6].

Eventually she was able to sign up at the Institution Protestant Deaconess at the Kairserswert in Germany for two weeks of training in July 1850 and again for three months in July 1851. It was then that she learned basic nursing, the magnitude of patient observation, and the value of good hospital organization. Through her social acquaintances, she became the Superintendent of the Institution for Sick Gentlewomen in Distressed Circumstances in London. Through her hardworking efforts, she realized that her services would be helpful in an institution where she would be able to train more nurses. She considered becoming the superintendent of nurses at King's College Hospital in London. However, politics, not nursing expertise, was to shape her next move [6].

Florence's father was a “landed gentleman” who set up a lead and weaving factory [7]. Florence Nightingale grew up as an upper middle-class woman in Victorian England [7]. She was one of the few ladies who were educated during Victorian England as a result of her father's opinion against the norm of the time that women of Nightingale's class must not attend university and also must not pursue professional careers but rather had to marry and bear children instead [8]. Due to her father's opinion against this norm, he single-handedly taught her Italian, Latin, Greek, philosophy, history, writing, and mathematics [8].

However, it must be noted that her father also arranged for a governess to teach her and her sister music and drama but her sister resented this decision [9]. From early childhood, Florence Nightingale got bored and frustrated as a result of the low-standard life that was reserved for women of her class in shrill contrast to that of the masses [10]. Florence had a strong will and this manifested in the “call” that she had which required her to devote her entire life to serving humanity at the age of seventeen [10].

Suzanne also commenting on Nightingale's “calling” indicated that

*“February 7, 1837 was a critical turning point in young Florence's life. She believed she heard the voice of God calling her to service. Following this mysticism, Florence became convinced God needed her for his service, but she remained baffled as to the exact job she was requested to do. Florence experienced much confusion and distress regarding her service to God. She travelled to Egypt and Greece in 1850 and engaged in spiritual discussions and thought as she travelled up the Nile” [11].*

Nightingale was eager to know what she will do with her calling and out of her contemplations she is believed to have said at a point in time that “today I am 30- the age Christ began his mission. Now Lord, let me only think of thy will, what Thou willest me to do-O-Lord, Thy will, Thy will” [11].

Upon returning from Egypt and Greece, Florence and her entourage visited Kaiserswerth in Germany, where Pastor Theodore Fliedner (1800–1864) had founded a hospital, orphanage, and school in 1836 [12]. At the age of thirty, Florence Nightingale returned to Kaiserswerth against her family's opposition to train as a nurse and she proved herself as a pupil with an outstanding ability after three months of training [12]. After her return from Kaiserswerth, she became idle for some time but, between 1851 and 1854, she put to use the practical experience that she gained by visiting hospitals throughout the United Kingdom and Europe to gather information [12].

Florence Nightingale's dream of becoming a proper nurse materialized when she came into contact with Sidney Herbert (1810–1861) during a winter holiday in Rome [9]. Sidney Herbert later became a foreign secretary for war and requested the service of Florence Nightingale to lead a team of thirty-eight nurses to assist at the main British Army hospital at Scutari, near Constantinople, to cure British wounded soldiers of the Crimean War [9]. However, Neuhaser has put forward that Nightingale went along with forty nurses and they helped clean up the wards [13].

Sidney Herbert's decision to appoint Florence Nightingale to lead the group of nurses was seen as a radical step because it was unprecedented in the sense that no woman had previously held an official position in the Army [12]. Upon their arrival, Nightingale and her team were faced with awful scenes as they were faced with dirty beds, clogged latrines, bad food, filth, and death [13]. For instance, in the week of 14 April 1855, 215 hand carts of filth were removed, the sewers were flushed 19 times, and the carcasses of two horses, a cow, and four dogs were buried by Nightingale's team of nurses [13].

Attewell has also put across that Nightingale was not wishing to imperil the prospect of alienating the doctors that she met at Scutari so she placed the nurses under the doctors' orders and within a month she secured the improvements in the upkeep of the wards, provided new bedding and clothing for the soldiers, and improved the hospital diets [12]. In addition to this claim, Attewell has also posited that Nightingale wrote letters on behalf of the soldiers to their families back at home, instituted a scheme for remitting moneys, and also provided reading rooms and games for the convalescents [12].

Cohen recounts the situation that Nightingale and her party of nurses met when they arrived at Scutari as follows:

*“The conditions Nightingale and her party found when they arrived at Scutari on November 5, the day of the major battle of Inkerman were appalling. The hospital barracks was infested with fleas and rats. Under the buildings, as a commission of inquiry later reported, ‘were sewers…loaded with filth…through which the wind blew sewer air up the pipes of numerous open privies into the corridors and wards were the sick were lying' on straw mats in a state of overcrowding that got even worse after Inkerman” [12]. *

Cohen has also noted that the canvas sheets, according to Nightingale, were “so coarse that the wounded men begged to be left in their blankets” and also the laundry was done in cold water which made the linens washed become “verminous” that they had to be destroyed [8].

Nightingale's spell at the Crimean was marked by many hardships. Among them is that “Florence Nightingale battled with the military authorities, the purveyors department and was a thorn in the side of the superintendent of Army medical services” [8].

C. J. Gill and G. C. Gill have also noted that there was a high case of corruption in the camp and this led to the siphoning off medical supplies; in order to curb this problem Nightingale established a parallel supply mechanism for critical materials and food, and this step made her prove that the official supplies were being stolen by sending her representatives to Turkish markets to buy back the purloined goods [14].

Nightingale in her own sense per the words of C. J. Gill and G. C. Gill, when she was faced with the imminent arrival of more patients, organized a team of 200 Turkish workers to replace the floor in Barracks hospital which was destroyed by fire and was an ideal habitat for fleas, flies, and lice [14]. Again, when Nightingale arrived at Scutari, she met deplorable Barracks which housed 10,000 sick men, with dirt and filth throughout the hospital. In addition, patients who were lying in the corridors and the wards were suffering from typhoid fever and cholera, as well as battling wounds [9].

In spite of this, Nightingale made a lot of impacts and contributions in her nursing adventure on Scutari. She instituted a lot of reforms, which were very essential. For instance, this is an account that a fever casualty brought to Scutari gave about the changes that he noticed, “everything changed for the better. The sick were not kept waiting in the passages but went at once to bed, were washed, and had clean linen and were attended as well as in England” [14].

Neuhauser has also pointed out that Nightingale recorded the outcomes of care which went a long way to helping her to estimate that the death rate among the patients was worst in February 1885 at 42.7% of all soldiers admitted [13]. She also instituted sanitary reforms which were started on 17 March 1855 and this reform caused the death rate to fall to 2.2% by June 1855 [13]. However, it must be taken into cognizance that, upon the reforms and the positive outcome that came out from it, the principal Medical Officer of the Army said that the reduction in death rates was due to the improved character of the cases coming from the Crimean peninsula [13]. Florence Nightingale however rebutted this claim by showing that the deaths were not due to battle field wounds but from infectious diseases and also went along to assert that the hospital's unsanitary environment led to the deaths of both wounded soldiers and healthy unwounded orderlies at Scutari [13].

After her service at Scutari, many are those who have associated Nightingale to be the originator of modern nursing and also had an enormous influence on contemporary nursing [14]. Among such influences is hospital infection control. C. J. Gill and G. C. Gill assess this influence in this way, “although the Crimean War settled nothing in terms of geopolitics, it served as the backdrop for a second struggle between the sanitarian movement and the medical dogma of the day, which the sanitarians at least won decisively” [14]. However, C. J. Gill and G. C. Gill were quick to note that “Nightingale cannot claim credit for originating the sanitarian theories, but the impact of her reforms in Scutari were so obvious and well publicized that the treatment of hospitalized and infected patients was forever changed” [14]. The second field influenced by Nightingale is hospital epidemiology. Nightingale was a skilled statistician and she was believed to be greatly influenced by the work of Adolphe Quetelet, the leading statistician of her days [14].

Her third influence on contemporary medicine is that on hospice medicine. Long before Kubler-Ross' theories about death with dignity, Nightingale has been noted to have practiced it in the sense that as a nurse she saved dozens of soldiers' lives but she also reported that she closed the eyes of hundreds [14].

### 3.1. Florence the Heroine in the Crimea War

 Bloy a Ph.D. Senior Research fellow at the Victoria Web conducted a research about the life of Florence Nightingale and the role she played in the Crimea War. According to Bloy's research in March 1854, the Crimea War broke out and the sufferings of the wounded soldiers created anger in Britain. Therefore, William Russell who was the Times correspondent published in the newspaper the state of wounded soldiers. Subsequently, Florence wrote to the war office offering her services on 14 October after her friend Sidney Herbert had written to her suggesting to her to go and help these wounded soldiers [15].

Bloy further argues that on 21 October Florence embarked on a journey to Crimea with thirty-eight nurses, ten Roman Sisters, eight Anglican Sisters of Mercy, six nurses from St. John Institute, and fourteen from various hospitals and they arrived on 4 November. She was the lady-in-chief [15]. Upon arrival, they saw the place to be very inappropriate to care for injured soldiers. Moreover, Bloy continues to argue that the military and the medical authorities at Scutari saw Nightingale's involvement as an indication on themselves [16].

Moreover, Bloy's research asserts that by the closing stages of 1854 Nightingale and her nurses had brought the Scutari hospital into an improved order. In December, other forty-six nurses went to add up to the other thirty-six. Through Florence hard work, she earned the name the “lady of the lamb.” As a result of the poor sanitation system at the Scutari hospital, there was an increase in Florence patients and this made the wards full and some died. In 1855, Florence fell ill from Crimea fever but she continued to work until the hospitals were closed and she returned to England in 1856. In September 1856, she visited Queen Victoria and Prince Albert at Balmoral and told them about the state of the military hospitals and reforms that need to be done [16].

Bloy further contends that in November 1853 the Nightingale Fund was set up to train nurses. By 1860 €50,000 had been collected and the Nightingale School and Home for Nurses was established at the St. Thomas Hospital. As a result of her health condition, she could not accept the post as superintendent but she watched the progress of the new institution and was able to use her experiences in Crimea for the assistance of nursing profession. She settled in London and lived a retired life of an invalid although she spent great deal of time offering counsel and support through her writings and also verbally. She also helped to set up nursing societies.

As a result of her hard working efforts, in 1901, she received the Order of Merit and in 1908 she was awarded the Freedom of City of London award. She had already received the German Order of the Cross of Merit and the French gold medal of Secours aux Blessés Militaires awards. On 10 May 1910, she was presented with the badge of honour of the Norwegian Red Cross Society [16].

Bloy's research on the role of Florence Nightingale shows that an extensive research was done about the lasting impacts Florence made in the Crimea War. However, Bloy's research entailed some shortcomings, among which was the failure to give an account of the cause of the Crimea War and also to highlight the parties involved in the war.

### 3.2. Florence Nightingale the Social Reformer

The Spartacus Educational Publishers researched about the social reforms made by Florence Nightingale after the significant role played in the Crimea War. The Spartacus Educational Publishers opine that, in order for Florence Nightingale to broaden her opinions on the reforms about the military hospitals, she published two books which were* Notes on Hospitals* (1859) and* Notes on Nursing* (1859). With the help from her affluent friends and John Delane at the Times, she was able to raise €59,000 to advance the quality of nursing. She used the money to start the Nightingale School and Home for Nurses at the St. Thomas Hospital. She also helped to train nurses for employment of workhouses [17].

Florence held an immense judgment about the rights of women therefore in her book* Suggestions for Thought to Searchers after Religious Truth*; she argued muscularly for the elimination of limits that prohibited women from having careers [17]. However, the Spartacus Publishers continue to argue that Nightingale powerfully contributed to the passing of Contagious Diseases Act. She preferred working behind the scene to get laws changed and disapproved of women making speeches in public [17].

Spartacus Educational Publishers support their assertion that women such as Elizabeth Garrett Anderson and Sophia Jex-Blake were dissatisfied by Florence's lack of support for women doctors. Nightingale argued that it was better to train nurses than women doctors. According to the Spartacus research on Florence biographer Colin Mathew emphasized that when Florence was sixty years, she considered herself old. Therefore, she tried to keep up with public health matters and continued to write corny addresses to probationers until 1889 but by now she was losing her eye sight. Therefore, she spent the whole of her fifteen years in her room in South Street and finally died in London on 13 August 1910 [17].

It is very important to note that the Spartacus Educational Publishers did an extensive research about the theme under review. However, Nightingale is known for the role she played in nursing and therefore the Spartacus Educational Publishers failed to throw more light on the reforms she made in the nursing profession.

Nightingale was a philanthropist from a wealthy English family who lived during a time when well-bred women from the upper class were not usually involved in caring for the sick. Despite the convention, Nightingale wanted to study the care and treatment of diseases and afflictions, so she enrolled in a three-month program to study nursing under the direction of Pastor Fliedner and his wife Erika at Kaiserswerth, Germany [18].

Upon graduation from the program, Nightingale became involved in creating organization called “Establishment for Gentle Women during Illness.” Ultimately, she was appointed to the leadership position of this organization, because she was knowledgeable as a result of prior experience in the administration of hospitals and she had expertise in nursing. As her work in nursing was acknowledged, she was consulted in the organization of training nurses; however, her efforts in Crimean War intervened. Florence Nightingale became involved in the Crimean War (1853–1856) after hearing about the squalid conditions of soldiers who had been injured. She organized other nurses who joined her in bringing aid, comfort, and supplies to injured soldiers.

When Nightingale returned home to London, she was honoured as a national heroine. She remained committed to establishing a program to train nurses. In 1860, Nightingale established a training program for nurses at St. Thomas's Hospital in London, where Florence Nightingale Museum is presently housed. Shortly thereafter, Nightingale took to her bed until her death in 1910. It was believed that her illness resulted from a weakened condition attributed to her work during Crimean War.

### 3.3. Nightingale and Advocacy

According to Macmillan English Dictionary, advocacy has been defined as a strongly public support for something. Sabatier and Weible identify coalitions built on advocacy as the essential tool to impacting changes in society and governmental programmes [19]. The scope of Florence Nightingale's effect on nursing and her usage of advocacy as a technical tool to operate are fundamental. “Modern nursing derives so completely from the examples and teachings of Florence Nightingale that is hard to pick out the particular practices that owe their existence to her influence. All nursing has been influence by her” [20].

However, Nightingale did not directly address the concept of advocacy. She demonstrated advocacy in exceptional ways throughout her lifetime. She did demonstrate in dramatic fashion in the Crimea that nursing and sanitation could reduce mortality. Nightingale was a singular force in advocating for as opposed to individuals, groups, and the nursing profession. Her expressions of advocacy grew with age, experience, and public acceptance of her as both nurse and expert. Her significant contributions include her advocacy for patient's environment connected to his or her health and advocacy in her leadership roles. Nursing is now recognizing how her ideas and techniques can be useful in the 21st century.

### 3.4. Advocacy through Patients Environment

“Nightingale environment theory stemmed from her core belief that nursing was in essence an act of using the environment to assist the patient in his or her recovery” [21].

Florence Nightingale was convinced that a patient's environment was connected to his or her health. She believed nurses could use sunlight, fresh air, pure water, cleanliness, and efficient drainage to allow nature to positively affect the patient's body, mind, and spirit, a revolutionary concept at the time. The year was 1854, and she was treating soldiers serving in the Crimean War. In those inhuman conditions, she believed that patients required a healthy mental, emotional, physical, and spiritual environment to raise their odds of recovery. She understood that physicians would treat the disease but nurses were the ones who could facilitate the healing environment by providing stimulating reading materials, emotional connections, and proper nutrition. According to Barbara Dossey, “Nightingale saw that nurses could be healing presence to patients, nurses should not go in angry and frustrate but convey a sense of calmness and intention.” Not only could nurses help influence the patient's environment to facilitate the healing recovery but they are also part of the environment. However, Nightingale saw that these conditions and practices from physicians and nurses could help improve the health conditions of the populace. That is to say, the importance of nursing's role in the management of the patient environment was a central theme she advocated for. She believed in* miasmatism*, the idea that foul odors caused disease. Although there is dispute as to the degree that the death rate was reduced in the Crimea, it is undeniable that there was a specific link between the state of the environment and the death rate.

### 3.5. Advocacy through Leadership

Leadership was one of Florence Nightingale's qualities. She offered emotional and spiritual comfort of care to the sick and the dying men as she walked down the dark hall with her lamp. These selfless practices earned her the nickname “Lady with the Lamp.” “Imagine a big hall that is three stories high and two football fields long, and in the dark these men would see Nightingale walking with her lamp to check on them, she was bring them a sense of hope and reassurance on a spiritual level” [21].

She also understood the importance of family to the sick thereby helping them to connect to their families back home. She helped the soldiers in the hospital write letters to their families because many could not read and write. “Nightingale was a change agent in her own right and seemed to be able to do without compromise; leadership techniques and advocacy were many of her strong points” [22]. She used leadership skills to her advantage in her tireless work with policy change in regard to human rights. She was able to change policy, making it possible for all faiths to be equally admitted to the hospital. It is very important for advance degree nurse, to be effective in her role as a leader and educator to provide students with the tools necessary to promote good healthcare.

Florence Nightingale's substantial legacy to health statistics is a composite of her accomplishments and her vision of what can and should be done by the profession. She wrote prolifically and demonstrated methods that were effective. Her lessons have become a roadmap for future generations. Most of her significant contribution to nursing is learned from her advocacy. Such advocacy includes the patient advocacy especially for the wounded soldier. Florence Nightingale created and built the nursing profession on the foundation of a holistic mind-body approach, assessing the individual and creating care plan interventions based on the emotional, psychological, medical, social, spiritual, and environmental challenges with the nurse being the primary advocate, fighting for and ensuring resources to maintain optimal outcomes and a decrease in mortality and morbidity, and also through showing great leadership qualities and the importance and value of nursing education.

At the end of the 19th century and the beginning of the 20th century, issues related to sanitation in relationship to the health of communities were primary concern of the healthcare planners and providers. During the 20th century, the discovery of new and more potent antibiotics and other scientific breakthroughs changed forever how the healthcare system dealt with infections. Towards the mid-20th century, a shift in priority from health of the community to the health and well-being of the individual occurred and towards the end of 20th century another shift toward care of the patient in the community occurred. Today, healthcare system is seen more sophisticated than previous generations because it is based on technology and science. Although such occurrence is happening in the 21st century, Florence Nightingale advocacy has so much influence on the management of healthcare worldwide.

### 3.6. Florence Nightingale's Advocacy on Egalitarian Human Rights and Leadership

The Online Journal of Issues in Nursing (OJIN) is a peer-reviewed, online publication that addresses current topics affecting nursing practices, research, education, and the wider healthcare sector. In an article written by Selanders and Crane in OJIN, attention was paid to how Florence Nightingale promoted advocacy through egalitarian human rights and leadership.

According to the research made by Selanders and Crane, Nightingale did not openly address the concept of advocacy. She rather verified advocacy in the outstanding ways throughout her lifestyle. This was demonstrated through her proceedings, ideas, and motivations through her communication [23]. Nightingale was a remarkable power in advocating for as opposed to individuals, groups, and the nursing profession. Her expression of advocacy grew with age, experience, and public reception of her nurse and expert. Her momentous contributions include her advocacy for egalitarian human right and her advocacy in her leadership role [23].

Selanders and Crane further argue that Florence in promoting advocacy through the backing of egalitarian human rights realized the lopsided status and opportunity given to men as compared to women in the English society. Therefore, in her aggravation she wrote a long essay named* Cassandra* in 1859/1979. In this book, she compares the professed value of a woman's activity to that of a man. Her first significant exhibition of advocacy for individuals came at the age of thirty-two. She assumed the position of a superintendent at the hospital for Gentlewomen in Distressed Circumstance, which was her first employment [23]. Although she had the support of the Ladies Committee, the body to which she reported, her first major apprehension was a policy held by the committee that only individual members of the Church of England would be admitted to the institution. Nightingale could not accept this position because of her liberal Unitarian upbringing and her beliefs in the value of individuals without the respect of religious fondness. Eventually she won the battle with the committee so that patients of all faith or no faith were equally admitted into the institution. She won this encounter partially through rational persuasion and also because she came from the upper class [23].

Selanders and Crane opine that, after this victory, Nightingale turned her attention to the expansion of care values for patients including the right to peaceful death. This was because the chronically and mentally ill were often disregarded by the hospital staff. Therefore, any staff member who did not work to Nightingale's standard was dismissed to signal and apply the administrative standards of care. They further maintained that Nightingale never wavered from the idea that the basic human right was high quality patient care that dedicated nursing staff should provide. This influenced Nightingale to advocate for patients on a larger stage during her twenty months in Scutari and the Crimea. Therefore, after her return to England, she established similar principles at the Nightingale School at St. Thomas Hospital [23].

The report of the researchers Selanders and Crane as found in OJIN on Nightingale advocacy through leadership also revealed that leadership was one of Florence Nightingale's intrinsic personas. Her education, social standing, wide range of connections, and global travel provided her with vital context, opportunity, and public voice which was used to promote her belief in the central role of the nurse to manage the environment [23]. Although this was an erroneous theory, it focused on the role of the environment in relation to illness. The deplorable conditions at Scutari reinforced this viewpoint, leading to her advocating for the importance of a suitable environment for patient both internally and extremely. She was also a supporter of the sanitation movement in London and therefore joins forces with reformers such as Farr and Chadwick in advocating for permanent improvement in public health. She saw that the expansion of nursing as crucial force would meet the growing healthcare needs in sectors outside the hospital [23].

The second major theme of leadership of Nightingale's advocacy accordingly was the establishment of the Nightingale School at St. Thomas Hospital in London, which was used to advocate for nurses who had a familiarity base and a specific role in healthcare. In this regard, she saw the expansion of nursing as an essential force for growing healthcare needs in sectors outside the hospital. Therefore, she resorted to the improvement of nursing in the military, midwifery, care of paupers, and nurse visiting (public health nursing). The Nightingale model of nursing education was executed through the following ways. Firstly, it was made to be firmly restricted to environment which included a nurse's home with a matron who served as a parent or guardian. Secondly, it extended nursing to the typical woman's sphere by projecting it as a domestic work that had been transplanted into the hospital [23]. The analysis above clearly shows that the OJIN did an extensive research on the Florence Nightingale as it discusses her two important advocacy areas.

### 3.7. Florence Nightingale's Philosophy of Nursing

Dr. Whitfield who is an adjunct faculty member in the online MSN program at Benedictine University conducted a research and published an article about the philosophy of Florence Nightingale to examine if present nursing has been able to meet the mark. Dr. Whitfield argues historical analysis of nursing practice over the years has helped to provide many examples of nurses' contribution to the communities. This raises the question on whether modern nursing has met or exceeded the standard set by Florence Nightingale. There is no doubt that Florence Nightingale is remembered for her stoicism (the stoics taught that distractive emotions resulted from error in judgment and that a sage or person of moral and intellectual perfection would not suffer such emotions) and significant impact on nursing today and also advocacy and commitment to her patients. Dr. Whitfield further argues that many of the criticisms levelled against Nightingale were because there was a false belief that she emphasized nursing as subservience to physicians or focused on the physical factors compared to psychological factors of nursing. Additionally, Dr. Whitfield maintains that there were many instances in Florence's life that contradict these beliefs. Nightingale understood very well the psychological connection to healing and actually believed that nurses should always speak up when things were unacceptable or inadequate [24]. She opines that Nightingale was an agent of change in her own right and seemed she was able to do so without any compromise. One of Nightingale advantages was her leadership techniques and advocacy for policy change, especially in the areas of human rights. Moreover, Dr. Whitfield calls on nurses of today to be effective in their roles as leaders and educators to promote health. Nurses should also be able to meet the needs of their patients without compromising their values and beliefs [24].

Dr. Whitfield concludes that the Nightingale's philosophy still rings loud and clear today and most likely continues to influence nursing and healthcare alike. Therefore, it would be much better if nursing today continues to enhance and build on Nightingale's theory thereby making the nursing profession an honourable one that exceeds the mark of Nightingale's philosophical legacy [24].

### 3.8. Evolution of Nursing Education in a Postindependence Context: Ghana from 1957 to 1970

The progress of nursing education in Ghana between 1957 and 1970 is described by vigorous change and progression [25]. The variations in nursing education arose within an economic climate that offered ongoing trials and a social and political climate that could be termed as neocolonial [25]. Ghana as a former colony to achieve independence first in Africa became the leader in the education of nurses which were undertaken at the University of Ghana to prepare tutors for the nursing profession [25].

Nursing education in Ghana prior to independence must be understood in the context of the organization of healthcare services by the colonial administrators [25]. Initially, these colonial masters suffered from tropical diseases like malaria, fever, and others that made the continent a graveyard but could not leave because of the profit they were getting from their trade. Therefore, an improvement of health conditions in the Gold Coast was a priority for Great Britain because of the rich mineral resources in the colony. The limited resources resulted in the focus of the medical officers to be restricted to taking care of the health of Europeans, African soldiers, and civil servants [25]. Consequently, The Gold Coast Medical Department was created in 1880, with responsibilities for preventative services such as vaccinations and sanitation [25]. Only about 2% of the Africans received treatments from this governmental health service and the majority sort treatment from traditional medical practitioners. Unfortunately, traditional healing was not recognized by the Europeans but, with time, they came to accept it as they gradually acknowledged the need to provide health service to the Africans. Subsequently, the British nursing sisters who came to Africa gave lessons to local nurses in human anatomy, physiology, surgical and medical nursing, and first aid [25]. Later, a maternity hospital was opened in Accra to recruit midwives through an ordinance for the establishment of midwifery training centre. The terms of the ordinance were in relation to examination, training, registration, and practice of midwifery in Gold Coast [25]. Until 1945, all senior nurses in Ghana, including nursing tutors, were white colonial sisters but the pattern changed with the years following the establishment of the centre [25].

At the time of independence, a lot of nursing education centres had been opened in Kumasi, Accra, Cape-Coast, and other places which offered training for state registered nurses (SRNs) and qualified registered nurses (QRNs), among others [25]. In 1958, a government decision to train doctors locally at the University of Ghana necessitated enlargement and modernization of Korle-Bu hospital to become a teaching hospital [25]. This decision had an impact on the evolution of nursing education and practice in Ghana and several SRNs were sent to UK to gain specialties in areas like orthopaedic, genitourinary, sick children nursing, and organization of central sterilizing departments in order for the nurses to function effectively within a teaching hospital milieu [25].

In an effort to strengthen nursing in Ghana, Marjorie Houghton, a former member of general nursing council in the colonial era, was invited by the government of Ghana to evaluate the nursing programs at the various training centres [25]. Several changes including the need for nurses to receive a four-year training, the discontinuation of prenursing course, and the requirement for student nurses to be supernumerary to ward stuff and the need for tutors and clinical instructors at the training centres were recommended [25]. Additionally, nursing education in Ghana made significant progress with the establishment of the first university program for nurses at the University of Ghana in 1963 [25]. This and many tremendous changes manifested in nursing education in Ghana.

## 4. Nurses and Nursing Education in Ghana: Creating Collaborative Opportunities 

Nursing education in Ghana is not different from the US and most African countries. While there are similarities, there are reflective variances as well. Globally, nurses play an essential role in healthcare provision scheme by stimulating health, preventing illness, restoring health, and lessening suffering [26]. However, worldwide, nursing has faced and continues to face noteworthy encounters to its personality and sustainability [26]. This trait is no different from nursing in Ghana. Nursing is a profession that is highly sought after although it is said there are not enough nurses due to one reason or another. In fact, there are not enough nurses in Ghana; the most highly educated have been leaving in large numbers to work in Great Britain or the United States [27].

In 2011, the Ghanaian Minister of Health hinted that Ghana lost nearly half of its nurses within a five-year period. He further stated that Ghana had only an estimated ten thousand nurses available to provide care to a population which was almost twenty million [27]. Sometimes, a condition under which some nurses or nursing is undertaken in the country is very appalling and this makes it difficult for the nurses to fully deliver their services efficiently especially in the rural areas. Most clinics in the country are many a time congested leading to poor services. There are some branches of nursing in the country that help ease the affliction of the citizens a bit. For instance, outreach clinics providing mostly medical staff comprised largely of nurses are an important component of the goal of the Ghanaian Ministry of Health (MOH) to reduce communicable disease and therefore, even with the difficulty and potential risk in travel, the nurses willingly go there [27].

Nurses are trained mainly to serve the public but they also help the nursing profession a lot. But the problem is once many of the nurses receive their certificate in public health, they prefer to travel outside and work instead of staying in the country to help [27]. A public health nurse must not only hold a nursing diploma but return to school for additional year of study; one can also leave school early and work as the equivalent of nurses' aide or a practical nurse at different times during their schooling [27]. The nursing profession is exposed to a high risk owing to direct contact with the bodily fluids of patients without any sort of protection [27].

There are different types of diseases in this country with the deadly HIV/AIDS reaching every aspect of life in Ghana. The incidence of AIDS has an overwhelming effect on the healthcare system, with an estimated five hundred thousand persons known to be infected with the disease, and the Ghanaian Ministry of Health projects that Ghana will experience nearly two million AIDS cases in the next ten years [27]. In this regard, our nurses became the main means of educating the public on health matters. Unfortunately, the pay and working conditions for nurses are very poor with limited chance for advancement in Ghana. [27]. Probably, these may be the reasons why some professional nurses travel to other countries to seek greener pastures.

### 4.1. Challenges to the Growth of Nursing Education in Ghana

Challenges are always issues that are encountered in every institution and nursing is no exception. Education for nurses in Ghana in the years following independence was fraught with problems for tutors and students alike; textbooks when available were frequently outdated and not within the financial means of the students [28]. Thus, nurses face financial issues both in terms of schooling and also after completion and working. Their salaries are not enough and this sometimes attracts strikes or quitting and seeking greener pastures abroad. Additionally, certain amenities needed for effective discharge of their duties are not available. For instance, common bed for patients to sleep on at times becomes an issue and the few available ones are in no better conditions. Also, clinical laboratories are poorly supplied and equipment is very old, and disposable nursing supplies are simply not available for teaching [26].

Management issue is another challenge of nursing in Ghana. There is lack of proper management in most of the nursing institution. Many a time, government hospitals are where these problems can be seen. The mere fact that the institution belongs to the government makes most nurses work unwillingly due to less supervision by management. Patients do not get the needed services that are required for them. The nursing profession in Ghana now has become more of a safe haven for unemployment issue since one is assured of her work after completion. This problem has generated some kind of bad nurses into the system since it is not with passion or interest that they went into the profession but rather to cater for their individual needs. Sometimes, situations of this sort found in most governmental hospitals are very alarming.

When nurses receive training, they are taught very well but as nurses leave the walls of their academic institutions, somehow many of them come out with the unwritten notion that the patient is expected to be ignorant about his or her health problem and the cure needed; the patient should be submissive to the health provider; and the patient should always be cooperative [28]. Nurses cannot carry such stereotyped ideas into the 21st century. The 21st century also comes with a rise in living standards, knowledge about patient rights, a highly educated population, and easy access to quality health information online [28]. Therefore, as countries explore new and innovative approaches to nursing practice in the 21st century, nurses should see all clients, both literate and illiterate, as partners in healthcare delivery and not as passive and subservient consumers [28].

### 4.2. Twenty-First Century Nursing in Kumasi and Its Environs

In an attempt to make a fair assessment of the twenty-first century Florence Nightingale in Ghana, the research called for the distribution of questionnaires to a number of nurses in Kumasi and its environs. Questionnaires were given to a total number of thirty (30) nurses, five (5) being males and the remainder twenty-five (25) females. The age range of respondents is between the ages of twenty (20) and forty (40) years. A question asked was “if the respondents chose the profession willingly”; in answering this question, twenty-six (26) answered in the affirmative while four (4) did not have it as their first choice. The professions of their choice were in the legal (2 respondents) and teaching (2 respondents) fields.

The reasons given for the choice of nursing as first option have been categorized. See Table 1.

Reasons given by respondents for settling into the nursing profession were as follows:

- The respect attached to the profession.
- Interest in the field.
- Motivated or inspired by someone in the field.
- Evading unemployment status.

The questionnaire further probed into the number of years the nurses have served in the profession.

Table 2 shows the distribution of nurses with regard to the number of years they have served. The number of people interviewed showed that a majority of the respondents have served within one month to two years; a period within one month to three years had seven nurses similar to the number of nurses that had also served within four to five years. Four of the respondents had served for a period above five years. However, two respondents failed to answer the question.

The respondents were also asked if they had faced any challenge in their profession. Sixteen answered positively and thirteen negatively and one failed to respond. The major challenges include nurses being exposed to health hazards due to lack of resources or logistics needed for service. Also the absence of needed facilities, lack of incentives, misconduct or attitude of some patients, delay of remuneration, and inadequate staff hands are other examples. Another issue raised was in relation to the time consuming factor of the profession which poses as a challenge to these nurses. This aspect will be addressed later in the discussion.

In response to the question “have you had any worse experience,” twenty-one (21) responded yes and three (3) no and six (6) failed to respond. Despite the fact that a significant number failed to respond, it can be deduced that majority of seventy percent (70%) had experienced unpleasant situations. Their experiences range from having to lose patients in the course of treatment, attending to accident victims or diabetic patients by treating wounds, needle pricks, inability to secure medication for patients when needed, patients attacking nurses (in one instance attempted rape), having to treat patients when doctors are on strike, and the difficulty of having to disclose results to patients (example HIV positive results). Other experiences include having to work without any form of remuneration, negative attitude displayed by some patients, and being sick on the job.

Due to the challenges faced by the respondents, it was further asked if they are willing to come to work each day. Ninety percent (27 people) were willing to go to work each day while ten percent (3 people) are not willing to go to work every day. It can be deduced from this data retrieved that several nurses in the Kumasi area and its environs have a sense of duty and are ready to take the practical steps to help save lives in Ghana and Asante in particular.

In relation to their numeration, it was surprising to note that there was a balanced equation in terms of response. Fifty percent (15 people) of the respondents were satisfied with their salaries while another fifty percent (15 people) were not satisfied. This makes it difficult to ascertain whether their salary or other forms of remuneration are able to cater for their needs or not and whether that had the tendency to affect their output at the work place.

Another question was asked to know whether nurses knew about the Hippocratic Oath. One was quick to note that nurses do not swear the Hippocratic Oath. Reasons assigned to the swearing of the oath are to help the nurses abide by rules and regulations of the profession, to be committed to the course, to exhibit good professional habits, to respect and honour their work, and to treat all patients equally. Twenty-six of the nurses responded that they abide by the oath; two said they did not; and another two failed to respond. This helps us to know that nurses are aware of their code of ethics and claim to act accordingly. A follow-up question was if they treat all patients equally. There was a one hundred percent response in the affirmative that they treat all patients equally. This assertion will be proven true or false in the course of the discussion in relation to nurses to patients' relationship.

The relationship that exists between nurses and patients was termed as cordial (15 people), good (5 people), and very good (2 people) and a person each described it as respectful, great, well, approachable, healthy, of good communication, humble, and professional.

Table 3 shows how patients treat nurses according to the data retrieved.

Table 4 shows that patients generally treat nurses well.

The majority of the respondents attend to an average of eleven to twenty patients per day, followed by an average of a patient to ten patients, more than fifty patients a day, twenty-one to thirty a day, forty-one to fifty daily, and the least number being thirty-one to forty patients daily. It can be deduced that the ratio of patients to nurses is higher. This therefore means nurses have an extra task of catering for numerous patients at a time. It also suggests that there are fewer nurses in the profession as compared to people who patronize the various hospitals within the Kumasi area and its environs.

Twenty (20) out of thirty (30) nurses responded that patients give them money before, during, or after treatment while the remaining ten claimed they had never received money from patients. Twenty-seven admitted that they did not expect patients to give them money; however, three deem it necessary. The question is whether it is ethically acceptable for nurses to receive gifts in the form of money items from patients before or after treatment? Would that necessarily serve as an incentive or a disincentive to the nurses in general? To what extent can that send a wrong signal to the general population or civil society in Ghana and Kumasi in particular? Did Florence Nightingale accept gifts before she attended to patients? The answer is a strong “no” as exemplified in the aforementioned literature on the service and contribution of Florence Nightingale. If the majority of her contributions to the treatment of the sick and the injured did not give credence to receiving of gifts from patients for whatever reason that might have come up, there is the need to focus on this as a form of Magna Carta in our discourse to improve the general nursing profession in Ghana and Kumasi in particular.

Table 5 is a reflection of the number of nurses that have dependants and how this affects their personal lives. Twelve (12) of the respondents have children; however, a significant number of nineteen (19) have relatives they cater for. This shows that majority of these nurses have dependants to cater for. Forty percent (40%) of the respondents claim their work affects their family life. The challenge of time factor could be associated with this phenomenon. Eighteen (18) on the other hand replied in the negative with regard to their work not affecting their family life. Seventeen (17) of the respondents did not have children and ten (10) of them did not have relatives they are taking care of.

Table 6 shows a distribution of nurses and their temperaments.

It can be deduced from the table that a lot of nurses are phlegmatic (33.3%), followed by nurses that are melancholic (30%), then followed by sanguines (26.7%), and the least being choleric (6.7%). This means that an insignificant number of choleric nurses are found in the health industry especially with regard to nurses in the Kumasi area. In the article “The Four Human Temperaments,” Erkstrand argues that the four basic human temperaments help us to more fully understand our basic behavioural disposition [29]. From scientific study and analyses, Erkstrand argues that all human personalities are commonly divided into four major categories with the exception of those with severe mental disorders and these four types are further split into two categories, that is, extroverts and introverts [29]. From the extroverted personalities that include the choleric and sanguine personality types are more outgoing, more sociable, and more comfortable in a crowd [29]. Also, the introverted personalities, that is, melancholy and phlegmatic personality types, are shyer and reserved and anxious about being in crowd, especially at being singled out in a crowd [29]. To an extent the kind of dominant temperament of an individual has the tendency or proclivity to shape his or her actions or behaviour at the work place. The essence for this discourse is to also ascertain how these nurses know their temperament and to what extent they are able to deal with the perceived or known weaknesses in their temperament. There is also the need to further assess how nurses who know their peculiarities or idiosyncrasies due to their temperament are able to dovetail that into their professional training to make them more functional to attain optimum performance at the work place.

The nurses were further asked if they would encourage their relatives to join the profession. Twenty-seven stated that they would encourage their relatives while three answered negatively. Reasons given for why they would encourage their relatives are based on humanitarian values, prestige, remuneration, communication skills, knowledge acquisition, inspiration, easy accessibility of employment, and increasing the number of staff. The general notion in Ghana is that nurses are well paid and that they are generally respected by the civil society in Ghana. Wearing a nursing school uniform in itself gives you prestige. The reasons given by respondents who would not encourage their relatives to join the profession include the fact that there are no incentives to motivate nurses and it is time consuming and very demanding.

Suggestions given by the nurses with regard to how the profession could be made better are as follows:

- Provision of logistics and resources.
- Respect for the profession.
- Increment of remuneration and other service allowances.
- Motivation and encouragement.
- Checking on attitude of patients and their relatives.
- Safety measures for workers.
- Increase in staff.
- Accommodation facilities for nurses.
- Provision of student allowance for nursing trainees.
- Research in the field.

In order to get a fair assessment and not a one-sided version, a total number of forty (40) patients were also engaged to answer some questions based on the output and attitude of nurses and the profession as a whole. The respondents consisted of twenty-eight (28) males and twelve (12) females.

Table 7 shows the age range of the respondents.

Patients prefer public hospitals to private hospitals and mostly use cash and carry as a mode of payment more than the National Health Insurance. This assertion was deduced after a frequency was created for the questions based on their preference in terms of the hospital they attend and the mode of payment.  Table 8 shows the raw figures of the data collected.

Table 9 shows the impression that most patients in Ghana have been using a sample size of forty. Majority of the respondents that is 57.5% indicated that the service provided by nurses is very dissatisfactory. Some of the reasons that accounted for this number include lack of ethics, inefficiency, lack of compassion and care for patients, disrespectful attitude, and lack of proper supervision, noncommitment, inadequate knowledge base, and laziness. However, eight respondents had positive remarks which include good services rendered, good behaviour exhibited by some nurses that can be described as kind and caring, and very encouraging services rendered as compared to a previous period. Another eight described their services as average, satisfactory, and improved but are impatient and have more room for improvement. This was categorized as neutral as they described the negative and positive sides of the coin.

Based on the assessment of the profession, a question was further asked if patients would allow their relatives to join the profession. Twenty-seven (27) responded yes while thirteen (13) responded no. The reasons given for encouraging relatives to join the profession were because it is a noble profession, humanitarian reasons, guaranteed employment after training, acquisition of skills and training, remuneration, impact making, and increasing the staff numeracy.

A question on whether any of the respondents have been a victim of maltreatment was asked and seventeen (17) responded “yes” while twenty-three responded “no.” Their response to this maltreatment includes feeling very bad, humiliated, angry, sad and confused, frustrated, unhappy, and astonished and finally becoming speechless. Education on code of ethics, counselling, aptitude test before recruitment, sanctions, and training; provision of awards or motivation; implementation of rules, improved logistics, effective supervision, and quality treatment of patients; increased wages; and periodic performance appraisal are the measures suggested by respondents to improve service delivery of nurses.

Also responses from patients concerning whether nurses take money or gifts after taking care of patients were not in the affirmative. The findings do not reflect the perception in Ghana that nurses generally take money or gifts from patients.

Patients believe that most nurses join the profession for recognition or prestige, passion or humanitarian sentiments, financial security, and guaranteed employment after school. With the question, will you entrust your life to a nurse, thirty-four of the respondents answered no while fifteen percent that is six people answered yes. This means that several of the patients in the respective communities do not have confidence in the nurses.

This is a detailed response given by respondents with regard to their worst experience with nurses. Their responses include “being ignored and not given immediate attention when needed,” “denied the privilege of visiting a family member,” “partiality and injection at the wrong place,” “wrong prescription,” “eating whiles I was in pain,” “vulgar language and favouritism,” “harsh words,” “shouting on patients,” “impatience,” “an injection that caused a two month pain,” “wrong language used,” “physical abuse,” and “conspiring with a patient to run away.”

There are mixed feelings with regard to how patients see the future of the nursing profession. Some of the respondents believe with time services rendered will improve while others believe it will deteriorate with time. The positive statements or phrases include “promising and bright,” “very good and bright,” “better services,” “a long way to save lives,” and “improvement in service.” Others also said that the profession needed effective supervision, more staff, the need to encourage people so that they do not lose trust in the service. Generally others hope for the best and see it as a means of secured employment. Others stated that there is the need for proper policy measures; otherwise the future will be bleak.

Services rendered by nurses are not encouraging as most of the responses from patients were negative more than positive. Nurses on the other hand believe that proper measures and availability of logistics and other resources will boost their morale for the job. The Florence Nightingale of the twenty-first century has her own challenges and this affects her output.

## 5. Discussion

Various writers contend that today's ailment forms show little modification from former centuries, yet the evolving signs with regard to morbidity and mortality present new tasks that must be met with new methods for better results. Moreover, for any nation to meet these tasks, it should have well-informed and capable nurses. These tasks often are indicative of wider matters of social, economic, political, and cultural setting of the people. It needs to be noted that no matter how difficult the tasks are, with the cooperative will of the people and the political will of the government, these tasks can be turned into prospects for solutions.

The analysis above has clearly laid bare effort of Florence Nightingale to provide the best care for patients in her days despite the challenges she encountered. The responses to the interviews granted to the nurses show clearly the challenges the nurses of today are facing in an effort to provide care for patients. In addition, the responses from patients who participated in the research present a vivid account of the kind of care and perception of healthcare delivery patients are receiving from the 21st century nurses. In spite of the similarities between the past and present, it can clearly be seen that the kind of care envisaged by Florence Nightingale for patients which she fought for is totally different from what majority of patients believed they are receiving in modern times. Therefore, it is highly recommended that the nurses of today in Ghana emulate the good deeds and spirit of Nightingale which was used to establish the nursing institution.

The study believes that solutions are not always simple. Therefore, suggestions on possible approaches to address these tasks are presented. We know of no universal solutions that exist to solve all the problems of healthcare delivery in all jurisdictions of the world. Health approaches must be addressed from various angles with specific results. It can be built on comprehensive information of the health needs and lifestyle patterns of the populace, tailored to local, socioeconomic, and political conditions.

These days with advancement in every aspect of life, technology, and globalization have impact on nursing and healthcare delivery. This is in the sense that since the world is a global market now, certain good practices of nursing can be adopted from other countries to help improve upon healthcare delivery in Ghana. We can look up to the rest of the world and adopt their good practices that are having great impact on their healthcare delivery in terms of personnel, management, and accommodation among many others. With technology, the country can now have better solutions to some illnesses that were previously not easy to diagnose and this can even help improve the life expectancy rate in the country.

Some of the ways to address this include the government making sure that finances of nursing are well taken care of so that they can live comfortably in order to provide efficient services to the nation. For a hospital or clinic to run effectively and efficiently, the availability of laboratory equipment which is in good conditions must be at the disposal of nurses. Those that are needed in teaching the students are also highly required.

It has been realized that international learning practice can offer outstanding prospect for nursing students to exercise nursing in cultural systems diverse from their own, gain an increased global outlook, and enhance their cultural capability. An international educational programme that engages students in healthcare in another culture is a wonderful way to immerse students in diversity issues. Therefore, Ghana needs an expansion of local nursing education and training capacity as well as well-coordinated international nursing education partnerships.

## 6. Conclusion

In conclusion, nursing in Ghana is prone to a lot of difficulties and challenges aside opportunities the job creates. With historical background of nursing available to us, we can look back and learn from the past, those practices that did not help nurses to function effectively and improve upon them. We can also know how to best address issues like finance, management, personnel, and many other issues that pose a threat to the nursing institution in an effort to keep the dreams and aspirations of Florence Nightingale, the great nurse that ever lived.

## Figures and Tables

**Table 1:** 

Category	Number of people
Being religious	2
Being inspired by people	3
Passion/dream	3
Empathy/humanitarian	9
Multiple reasons	8
No answer	1
Total	26

**Table 2:** 

Years served in the profession	Number of people
1 month–2 years	10
2 years 1 month–3 years	7
4 years–5 years	7
Above 5 years	4
No answer	2
Total	30

**Table 3:** 

Good/positive	Bad/negative	Neutral	No answer
23	1	4	2

**Table 4:** 

Number of patients treated daily	Frequency of nurses
1–10	7
11–20	8
21–30	4
31–40	2
41–50	3
More than 50	6

**Table 5:** 

Question	Yes	No	No answer
Does your occupation affect your family life?	12	18	—
Do you have child/children?	12	17	1
Do you have relatives that you look after?	19	10	1

**Table 6:** 

Temperament	Number of respondents
Sanguine	8
Choleric	2
Melancholic	9
Phlegmatic	10
No answer	1

**Table 7:** 

Age range	Number of patients
11–20	8
21–30	23
31–40	1
41–50	2
Above 50	1
No response	5
Total	40

**Table 8:** 

Questions	Options	Number of people
Which hospital do you prefer to attend?	Public hospital	24
	Private hospital	16

Which mode of payment do you use?	Cash and carry system	20
	Insurance	19
	No answer	1

**Table 9:** 

Impression	Number of people
Negative	23
Positive	8
Neutral	8
No answer	1
Total	40
